# Peptide-MHC-targeted engineered virus-like particles enable selective priming and gene editing of tumor-specific T cells

**DOI:** 10.1016/j.celrep.2026.117510

**Published:** 2026-06-09

**Authors:** Brian H. Shim, Q. Henry Zhao, Jack A. Queenan, Blake E. Smith, Michael E. Birnbaum, David R. Liu

**Affiliations:** 1 Merkin Institute of Transformative Technologies in Healthcare, Broad Institute of Harvard and MIT, Cambridge, MA, USA; 2 Department of Chemistry and Chemical Biology, Harvard University, Cambridge, MA, USA; 3 Howard Hughes Medical Institute, Harvard University, Cambridge, MA, USA; 4 Department of Biological Engineering, Massachusetts Institute of Technology, Cambridge, MA, USA; 5 Koch Institute for Integrative Cancer Research, Cambridge, MA, USA; 6 Ragon Institute of MGH, MIT and Harvard, Cambridge, MA, USA; 7 Singapore-MIT Alliance for Research and Technology Centre, Singapore, Singapore; 8 These authors contributed equally; 9 Lead contact

## Abstract

Tumor-infiltrating lymphocyte (TIL) therapies harness tumor-specific T cells endogenous to a patient’s repertoire but their efficacy is limited by challenges such as low frequencies of tumor-specific clonotypes and dysfunctional T cell phenotypes. These challenges necessitate technologies to engineer and reprogram endogenous tumor-specific TILs *ex vivo*. Here, we present a strategy using engineered virus-like particles (eVLPs) pseudotyped with peptide-major histocompatibility complexes (pMHCs) as a programmable, single-effector platform for selective and coordinated priming, expansion, and genome editing of rare antigen-specific CD8^+^ T cells among their endogenous polyclonal repertoires. We demonstrate that pMHC-pseudotyped eVLPs (pMHC-eVLPs) deliver T cell function-enhancing base editors to arm polyclonal lymphocytes with enhanced anti-tumor cytotoxicity by selectively expanding and engineering the tumor-specific T cell compartment. Our work establishes pMHC-eVLPs as a platform for enhancing TIL therapy with precision gene edits without the risks of bystander T cell engineering associated with polyclonal TIL engineering approaches.

## In brief

pMHC-pseudotyped engineered viruslike particles (pMHC-eVLPs) enable coordinated priming, expansion, and precision gene editing of rare antigenspecific CD8^+^ T cells populations among their endogenous repertoires while leaving the bystander T cell repertoire unmodified. pMHC-eVLP-mediated T cell reprogramming arms polyclonal lymphocytes with enhanced anti-tumor cytotoxicity by selectively expanding and engineering the tumor-specific T cell compartment.

## Graphical Abstract

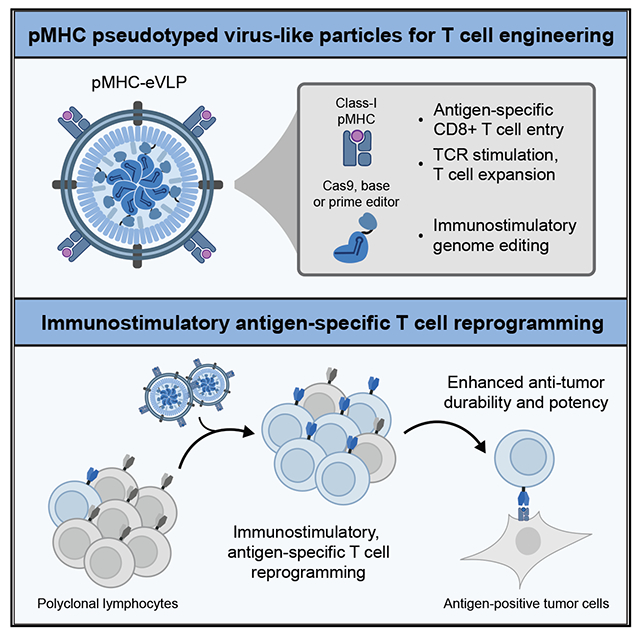

## INTRODUCTION

The goal of cancer immunotherapy is to stimulate robust immune responses against malignant cells while sparing healthy tissues and therefore requires immune targets that are cancer-specific. Immunogenic peptide antigens displayed by class I major histocompatibility complexes (MHC) on the surface of tumor cells, such as tumor-associated antigens, cancer-testis antigens, and neoantigens, represent a promising such class of targets to direct precision cancer immunotherapy. Tumor-infiltrating lymphocyte (TIL) therapy, a form of adoptive cell therapy, harnesses the potential of endogenous T cells found within tumors to target such tumor-restricted antigens. TIL therapies are produced by isolating autologous T cells from tumor resections and extensively expanding them *ex vivo* prior to reinfusion into the patient.^1^ Early investigations of TIL therapy demonstrated an ability to drive durable remission in metastatic melanoma; since then, the detection of both TILs and neoantigens in a range of other cancers has corroborated the potential of TIL therapy for treating multiple cancer types.^2–7^ However, TIL products face several challenges that limit their efficacy. Only a fraction of TILs are tumor-specific, and TILs often exhibit exhausted, dysfunctional, or weak effector phenotypes induced by chronic stimulation and immunosuppressive conditions within the tumor microenvironment. These limitations impede the ability of many TIL products to mount aggressive and durable anti-tumor responses.^8–11^

Recent efforts have used precision gene editing strategies and synthetic genotypes that equip therapeutic T cell products with enhanced antigen sensitivity, improved potency and durability, and the ability to withstand immunosuppressive conditions while circumventing dysfunction.^12–18^ However, current methods can only introduce these function-enhancing genotypes nonspecifically to broad, polyclonal T cell repertoires. This lack of specificity poses the risk of enhancing bystander T cells, which are abundant in most TIL cultures, increasing the potential for undesired off-target immune toxicity. The inability to install immunostimulatory gene edits selectively in the tumor-specific T cell compartment of TILs therefore impedes the clinical application of these gene editing strategies to enhance TIL therapy.

We envisioned that a programmable, single-effector strategy capable of activating, expanding, and gene editing user-defined antigen-specific CD8^+^ T cells could enhance the composition, phenotype, and efficacy of therapeutic TIL products. Here, we describe the development of a therapeutic strategy using peptide-MHC-targeted engineered virus-like particles (pMHC-eVLPs) that enables highly selective and coordinated priming, expansion, and editing of rare antigen-specific CD8^+^ T cell populations among their endogenous repertoires while leaving the bystander T cell repertoire unmodified. We demonstrated that pMHC-eVLPs can be readily equipped with diverse peptide antigens presented in various HLA allotypes and applied them to edit T cells specific to multiple clinically relevant tumor antigens. We engineered multiple aspects of the pMHC-eVLP particle to achieve therapeutically relevant delivery efficiencies in primary cells, and applied optimized pMHC-eVLPs to functionally reprogram polyclonal lymphocyte expansions *ex vivo* for enhanced tumor control. Our findings establish pMHC-eVLPs as a novel agent for cancer immunotherapy that can enhance TIL therapy without the risk of unwanted bystander T cell engineering.

## RESULTS

### eVLPs can edit and reprogram primary human T cells

Forward genetic studies using CRISPR-based gene perturbation screens have enabled the discovery of precision gene editing strategies that can modulate T cell function and enhance T cell therapies.^12–18^ Recent reports have described nuclease- and base editor-mediated genetic modifications that can enhance T cell metabolism, antigen sensitivity, durability under immunosuppressive conditions, and anti-tumor cytotoxicity.^12–18^ We recently developed eVLPs, which lack viral genomes and instead package non-native protein cargoes, as a method for therapeutic delivery of gene editor ribonucleoprotein (RNP) complexes to living cells *in vitro* and *in vivo.*^19–21^ Because eVLPs are enveloped by their producer cell’s membrane, their viral surface can be readily functionalized with non-native or chimeric envelope proteins to modulate target cell tropism.

**Figure 1.**
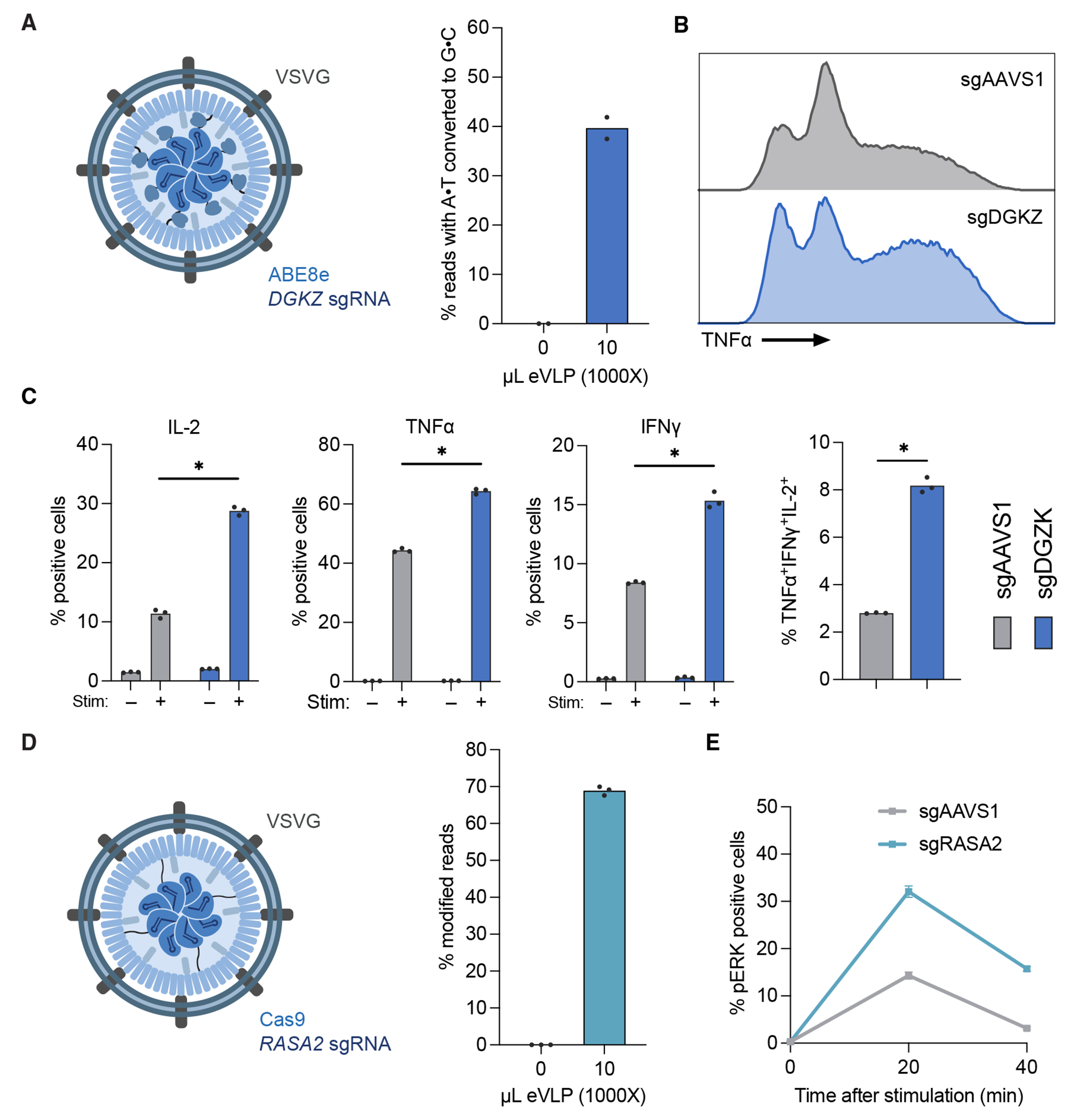
Primary human T cell engineering with eVLPs (A) Schematic of ABE-eVLP and base editing efficiency for *DGKZ* L378P in primary human T cells following eVLP treatment. (*n* = 2 replicates). (B) Representative flow cytometry histograms of TNFα in edited T cells following stimulation. (C) Percent positivity for the indicated intracellular stains in edited T cells following stimulation. *p* values calculated using unpaired t test. *p < 0.05. (*n* = 3 replicates). (D) Schematic of Cas9-eVLP and modification frequency at *RASA2* in primary human T cells following eVLP treatment. (*n* = 3 replicates). (E) Percent positivity for phosphor-ERK in edited T cells at the indicated time intervals following stimulation. (*n* = 3 replicates).

We first assessed whether non-targeted eVLPs displaying the broadly tropic fusogen, vesicular stomatitis virus glycoprotein (VSVG), could be used to functionally reprogram primary human T cells by installing immunostimulatory gene edits. We produced eVLPs packaging the adenine base editor ABE8e and a guide RNA (sgRNA) designed to install a synthetic gain-of-function allele (L378P) in *DGKZ*, a negative regulator of T cell activation.^14^ High-throughput sequencing (HTS) of T cells treated with these ABE-eVLPs revealed efficient installation of the desired L378P allele at *DGKZ*, and *DGKZ* base-edited T cells exhibited an enhanced effector cytokine response to antigen stimulation compared to control edited cells, consistent with previous reports^14,16^ (Figures 1A–1C; Figure S1A). Next, we transduced T cells with eVLPs packaging Cas9 nuclease and an sgRNA targeting *RASA2*, a checkpoint of T cell antigen response signaling.^12^ HTS analysis revealed efficient *RASA2* ablation in Cas9-eVLP-treated T cells, and *RASA2*-edited cells displayed enhanced signaling downstream of antigen stimulation compared to control edited cells, consistent with previous reports^12^ (Figures 1D and 1E; Figure S1B). Finally, we explored the ability of prime editor eVLPs (PE-eVLPs) to rewrite two adjacent codons in *IL2RB* (H133D, Y134F) to convert wild-type IL-2 receptor-expressing T cells into orthogonal (*ortho*) *ortho*IL-2R T cells that respond to *ortho*IL-2 but not wild-type IL-2 cytokine.^22^ We produced eVLPs packaging PEmax+SSB prime editor and an optimized pegRNA and nicking guide RNA (ngRNA) pair, and found that PE-eVLPs were capable of installing this challenging prime edit in primary T cells (Figures S2A and S2B).^23^

Taken together, these data demonstrate that eVLPs can be used to efficiently edit primary human T cells with base editors, nucleases, and prime editors, and that eVLP-mediated immunostimulatory gene editing can arm T cells with enhanced effector function.

### Targeted gene editing of antigen-specific T cells with pMHC-eVLPs

Reprogramming the tropism of therapeutic viral vectors is an established strategy in which a desired cell-type-selective targeting molecule is paired with a viral glycoprotein to mediate cell entry or endosomal escape. Recent efforts have leveraged an endosomal fusion-competent but receptor-blind, mutant form of VSVG, VSVGmut, to enable intracellular cargo delivery by vectors displaying natural ligands or antibody-derived single-chain variable fragments (scFvs) that target cell-type-specific cell surface proteins.^24–26^ The use of scFvs targeted to CD3, an invariant component of T cell receptor (TCR) complexes, has been described as a method to direct the tropism of lentiviruses to CD4^+^ and CD8^+^ T lymphocytes broadly.^27,28^

**Figure 2.**
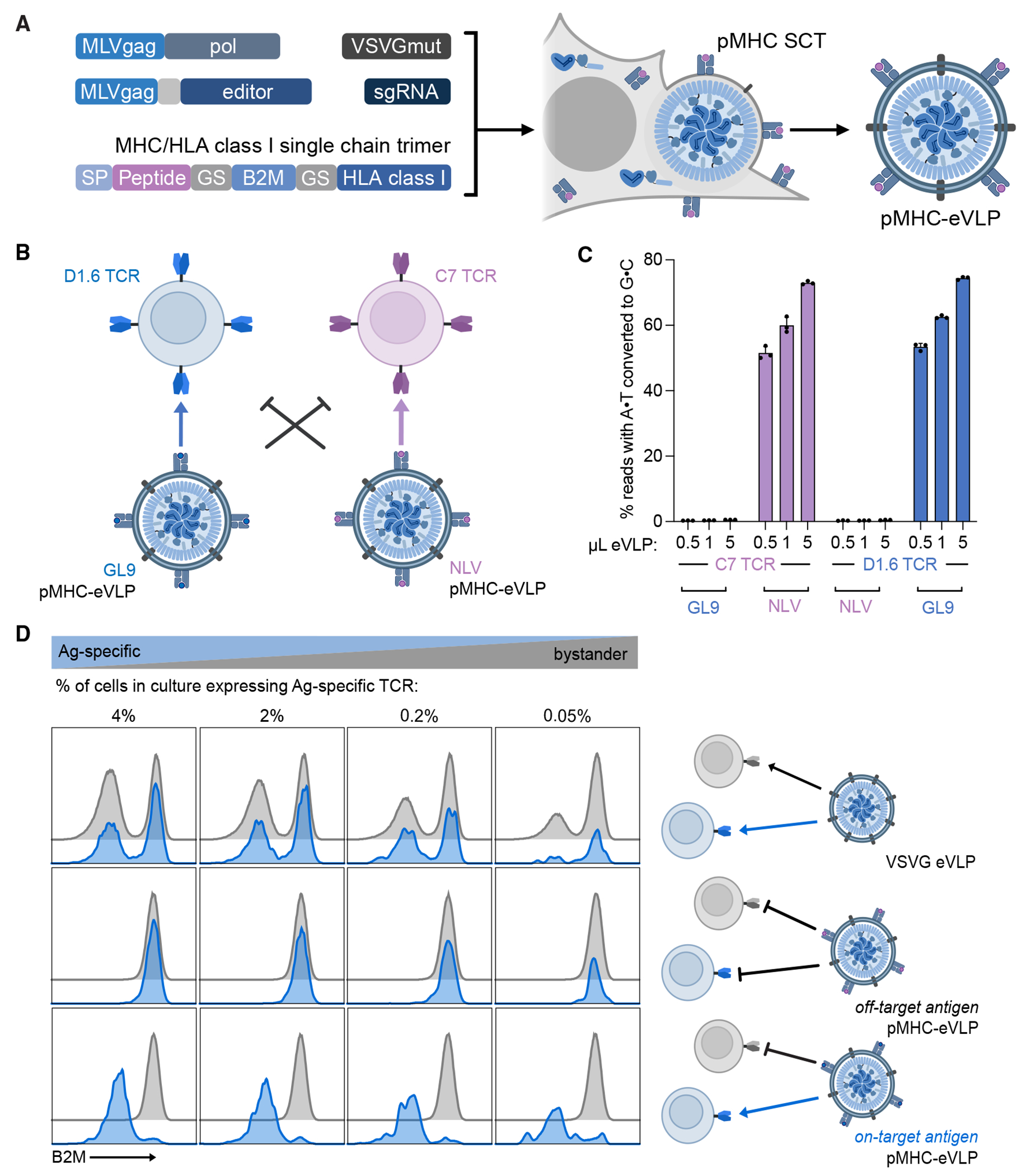
Targeted gene editing of antigen-specific T cells with pMHC-eVLPs (A) Schematic of pMHC-eVLP composition and production. (B) pMHC-eVLP selective transduction experiment using clonal TCR-expressing Jurkat cells. (C) Base editing efficiencies at *B2M* in the indicated TCR cells treated with pMHC-eVLPs displaying the indicated antigens. (*n* = 3 replicates). (D) Representative flow cytometry histograms of B2M expression in cell mixtures. Columns represent the indicated on-target cell frequencies and rows represent eVLPs for treatment. Blue populations are gated for on-target antigen-specific cells and gray histograms are gated for off-target bystander cells according to cell-tracking dye.

While CD3-targeting strategies can reprogram RNP delivery vector tropism to polyclonal CD4^+^ and CD8^+^ T cells, we sought to develop a more selective delivery strategy for antigen-specific CD8^+^ T cells given the lack of existing methods to specifically engineer tumor-specific cytotoxic T cells with precision gene editing. We previously demonstrated that lentiviruses co-pseudotyped with class I peptide-MHCs (pMHCs) and VSVGmut can direct transgene integration selectively to antigen-specific T cells, and applied this retroviral strategy to map the antigen specificity of polyclonal human T cell repertoires and to deliver immunomodulatory transgenes to antigen-specific murine T cells for therapeutic T cell engineering.^25,29^ However, it is well-established that permanent integration of transgenes encoding gene editing agents poses significant genotoxic risks associated with permanent editor expression, such as increased off-target editing, chromosomal aberrations, and genomic instability.^19,20,30,31^ We hypothesized that a similar pMHC pseudotyping strategy could be adapted to reprogram eVLP tropism—and therefore transient gene editor RNP delivery—to enable selective and efficient precision editing of antigen-specific human CD8^+^ T cells while circumventing the risks associated with permanent integration of transgenes encoding gene editing agents by lentiviruses (Figure 2A).

To test this approach, we generated ABE8e-eVLPs displaying class I pMHC single chain trimers (SCTs) presenting one of three epitopes derived from viral proteins in the context of HLA-A*02:01: GL9 (GILGFVFTL) from influenza A matrix protein, NLV (NLVPMVATV) from cytomegalovirus (CMV) protein pp65, or GLC (GLCTLVAML) from Epstein-Barr virus (EBV) protein BMLF1 (Figures 2B and S3A). We used these pMHC-targeted eVLPs (pMHC-eVLPs) to treat Jurkat T cells expressing TCRs with known specificity to each antigen: D1.6, C7, and D2.4, respectively^32^ (Figures 2B; S3A). Strikingly, we observed eVLP transduction and adenine base editing at the target *B2M* locus exclusively in T cells treated with pMHC-eVLPs displaying the cognate epitope to its TCR, with no detected editing in T cell clones treated with eVLPs displaying a non-cognate antigen (Figures 2C; S3B–S3D), confirming that pMHC-eVLPs could be used as selective gene editing agent delivery vehicles to antigen-specific T cell clones. We additionally confirmed that pMHC-eVLPs could be programmed to deliver cytosine base editor and prime editor RNPs for efficient cytosine base editing and prime editing of antigen-specific T cells (Figures S3E and S3F).

Having demonstrated the ability to selectively edit clonal T cell populations in an antigen-specific manner, we next explored whether pMHC-eVLPs could selectively target rare antigen-specific T cells among complex T cell mixtures. We labeled D1.6 TCR-expressing Jurkat T cells with a cell-tracking dye and mixed them with unlabeled T cells expressing irrelevant TCRs, treated these cell mixtures with *B2M*-ablative ABE8e-eVLPs, and assessed B2M expression via flow cytometry as a measure of eVLP transduction following treatment. We observed that GL9 pMHC-eVLPs efficiently and selectively knocked down B2M expression in cognate D1.6 T cells but not bystander T cells, even when target D1.6 T-cells were as rare as 0.05% (Figure 2D). In contrast, VSVG eVLPs transduced all T cells indiscriminately, whereas off-target NLV pMHC-eVLPs did not measurably transduce any T cells (Figure 2D).

Taken together, these results establish pMHC-eVLPs as a viable platform for selectively delivering base editor RNPs to rare antigen-specific T cell clonotypes but not bystander T cells with irrelevant antigen specificities.

### pMHC-eVLPs selectively target tumor-specific T cells

**Figure 3.**
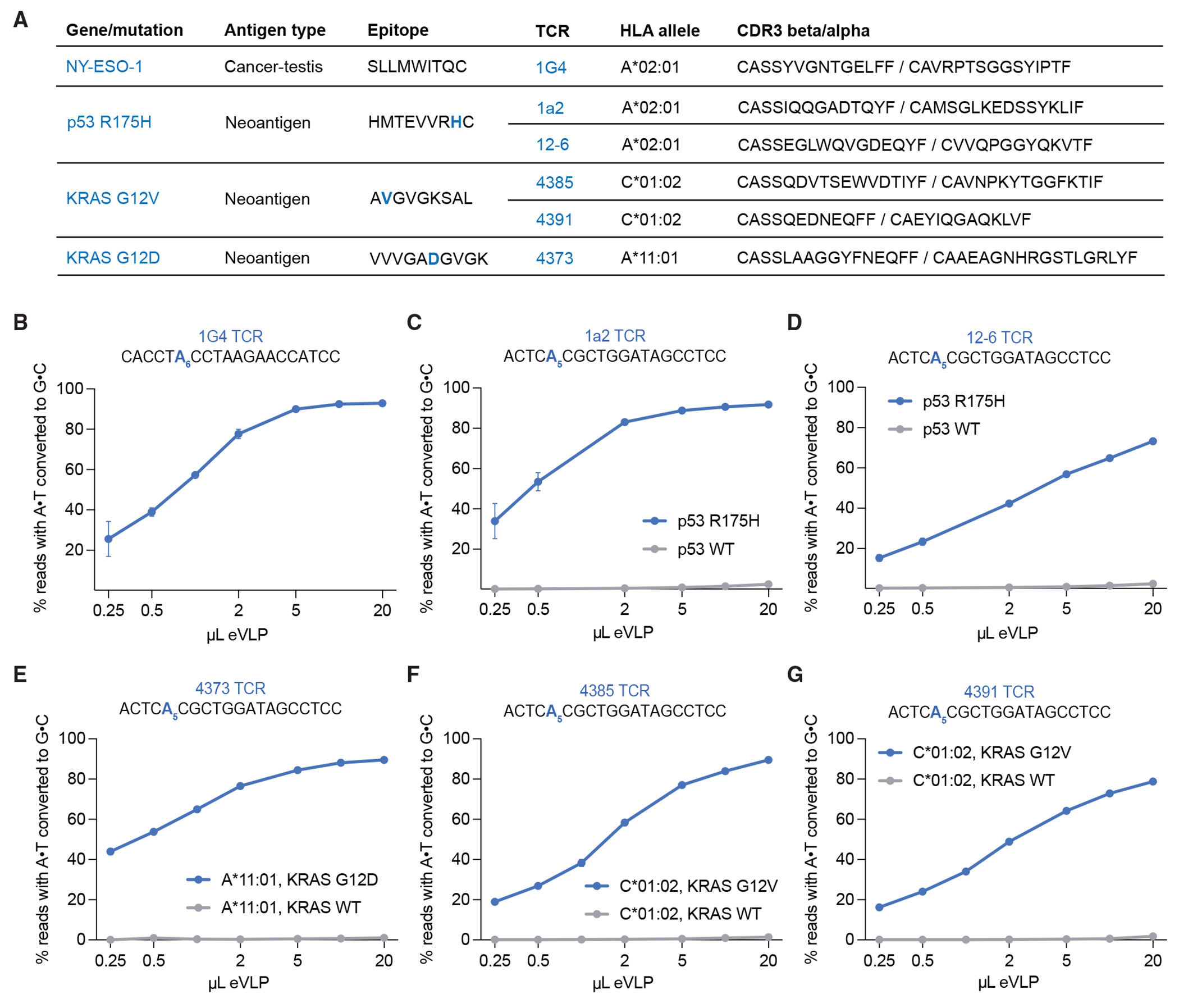
Tumor-specific T cell editing with pMHC-eVLPs (A) Table of tumor antigens, HLA alleles, and cognate tumor-specific TCRs used. (B–G) Base editing efficiencies in clonal tumor-specific TCR-expressing Jurkat cells by pMHC-eVLPs displaying relevant tumor or control antigens. (*n* = 3 replicates).

Having demonstrated the ability to target and edit antigen-specific T cell clonotypes using viral model antigens, we next explored the translatability of the pMHC-eVLP platform for targeting T cell clonotypes specific to therapeutically relevant tumor antigens. We first targeted Jurkat T cells expressing the 1G4 TCR, which is specific to the cancer-testis antigen NY-ESO-1_157_–_165_ in the context of HLA-A*02:01, and found that eVLPs displaying NY-ESO-1_157_–_165_/HLA-A*02:01 could efficiently edit cells bearing cognate 1G4 TCRs^33^ (Figures 3A and 3B). We next targeted Jurkat T cells with engineered specificity to public cancer neoantigens derived from shared p53 R175H, KRAS G12V, and KRAS G12C hotspot mutations presented in the context of HLA allotypes HLA-A*02:01, HLA-A*11:01, and HLA-C*01:02^34,35^ (Figure 3A). pMHC-eVLPs displaying neoantigens were able to edit cognate neoantigen-specific TCRs with high efficiency, whereas pMHC-eVLPs displaying unmutated self-peptides were inert against anti-tumor TCRs (Figures 3C–3G), confirming that pMHC-eVLPs can be reprogrammed to target T cell clonotypes with therapeutically relevant anti-tumor specificities.

We hypothesized that the affinity of a given TCR·pMHC interaction might influence eVLP delivery efficiency. To test this relationship, we treated 1G4 T cells with pMHC-eVLPs bearing a series of NY-ESO-1 antigen mutants with characterized affinities for the 1G4 TCR^36^ (Figure S4A). We observed that the transduction efficiency of pMHC-eVLPs displaying the mutant epitopes strongly correlated with TCR·pMHC affinity, indicating that TCR-mediated transduction of pMHC-eVLPs is in part determined by the relative strength of the specific TCR·pMHC interaction (Figures S4B and S4C). Importantly, TCR·pMHC Kd values ranging from 7.94 to 90.8 μM supported robust eVLP transduction. These data suggest that the pMHC-eVLP platform may be broadly applicable for CD8^+^ T cell delivery via natural TCRs, which typically span Kd values of 1–100 μM to their cognate class I pMHC,^37^ and does not require super-physiological TCR·pMHC affinities to achieve targeted genome editing.

We confirmed that pMHC-eVLPs form functional TCR·pMHC interactions that productively activate TCR signaling during transduction, observing acute upregulation of both CD69 and NFAT following antigen-specific pMHC-eVLP treatment (Figures S4D and S4E).^25^ We found that this productive TCR signaling is important for TCR-mediated pMHC-eVLP transduction, as inhibition of downstream TCR signaling by dasatinib highly attenuated T cell transduction by antigen-specific pMHC-eVLPs but did not affect TCR-independent transduction by VSVG-pseudotyped eVLPs (Figure S4F).^25^

We additionally asked whether the formation of catch-bonds versus slip-bonds between certain TCR·pMHC pairs might influence antigen-specific pMHC-eVLP transduction. Catch-bond formation has been shown to enable robust T cell activation for even low affinity TCR·pMHC interactions.^38^ We therefore generated Jurkat T cells bearing TCR55, which forms a weakly activating slip-bond with the HIVpol/B*35 pMHC but a highly activating catch-bond with the Pep20/B*35 pMHC despite comparable and physiological TCR·pMHC affinities,^38,39^ as well as T cells bearing an engineered TCR55-A50E, which forms catch-bonds with both HIVpol/B*35 pMHC and Pep20/B*35 pMHC. We treated TCR55 and TCR55-A50E cells with pMHC-eVLPs pseudotyped with either HIV/B*35 or Pep20/B*35 and subsequently assessed T cell activation by CD69 upregulation and eVLP transduction. Consistent with previous reports, we observed increased CD69 upregulation in TCR55 cells by Pep20/B*35 eVLPs compared to HIV/B*35 eVLPs, whereas TCR55-A50E cells were similarly activated by both pMHC-eVLPs (Figure S4G). Notably, pMHC-eVLPs presenting HIV/B*35 were still able to induce CD69 upregulation on TCR55 cells despite reported slip-bond formation (Figure S4G). Interestingly, both HIV/B*35 and Pep20/B*35 pMHC-eVLPs were able to efficiently transduce both TCR55 and TCR55-A50E cells (Figure S4H), suggesting that antigen specificity and physiological TCR·pMHC affinity, rather than catch-bond formation, are the primary determinants of pMHC-eVLP-mediated T cell activation and transduction.

Collectively, these findings demonstrate that the pMHC-eVLP platform can be programmed with different antigens and HLA allotypes to enable selective and efficient targeting of tumor-specific T cells via natural TCRs with physiological antigen affinities, and that pMHC-eVLPs induce antigen-specific T cell activation during TCR-mediated entry.

### pMHC-eVLPs enable editing and expansion of primary antigen-specific T cells

**Figure 4.**
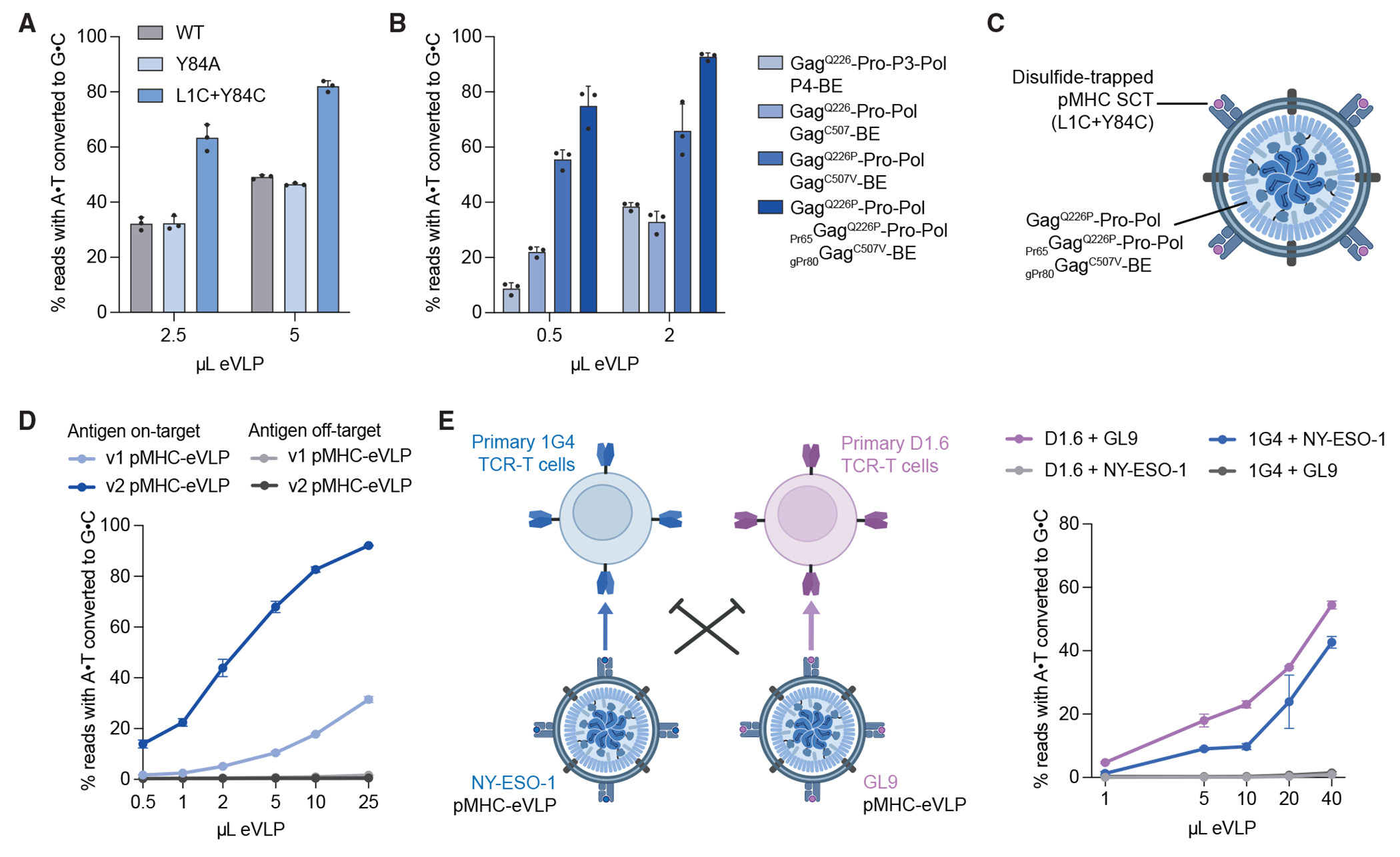
Engineering of enhanced pMHC-eVLPs (A) Base editing efficiencies in 1G4 TCR Jurkat cells by NY-ESO-1 pMHC-eVLPs bearing the indicated SCT mutations. (*n* = 3 replicates). (B) Base editing efficiencies in 1G4 TCR Jurkat cells by NY-ESO-1 pMHC-eVLPs produced using the indicated capsid architectures. (*n* = 3 replicates). (C) Schematic of optimized v.2 pMHC-eVLP architecture. (D) Base editing efficiencies in 1G4 TCR Jurkat cells by v.1 or v.2 pMHC-eVLPs displaying on-target or off-target antigens. (*n* = 3 replicates). (E) Base editing efficiencies in primary TCR-T cells bearing the indicated TCRs by v.2 pMHC-eVLPs displaying the indicated antigens. (*n* = 3 replicates).

Next, we engineered the initial (v.1) pMHC-eVLP particle to improve delivery efficiency. Given that pMHC displayed on the eVLP surface is critical for targeting function, we first incorporated mutations into the pMHC SCT previously reported to stabilize the chimeric complex. We found that a “disulfide-trapped” pMHC mutant (L1C, Y84C) that further stabilizes the covalently linked peptide in the MHC groove^40^ markedly improved selective transduction efficiency into target cells (Figure 4A). Next, we tested a series of engineered and evolved eVLP variants to identify an optimal capsid and RNP cargo-loading architecture, and found that particles produced using the evolved “v.5” gag capsid mutants in the MMLV gPr85 Gag polyprotein were optimal for selective pMHC-eVLP delivery efficiency^19,21,41,42^ (Figure 4B). Combined, these optimizations yielded an optimized v.2 pMHC-eVLP particle with significantly improved delivery efficiency compared to our initial v.1 design while maintaining high antigen-specific selectivity (Figures 4C and 4D).

We next tested whether optimized v.2 pMHC-eVLPs were capable of selectively transducing primary human T cells bearing TCRs with cognate antigen specificity. We produced TCR-T cells bearing either 1G4 or D1.6 TCRs using primary T cells isolated from healthy human donors and treated these antigen-specific TCR-T cells with either pMHC-eVLPs displaying NY-ESO-1 or GL9 antigen. Indeed, we observed that v.2 pMHC-eVLPs were capable of efficient primary T cell transduction in a highly antigen-specific manner, consistent with our earlier experiments (Figure 4E).

**Figure 5.**
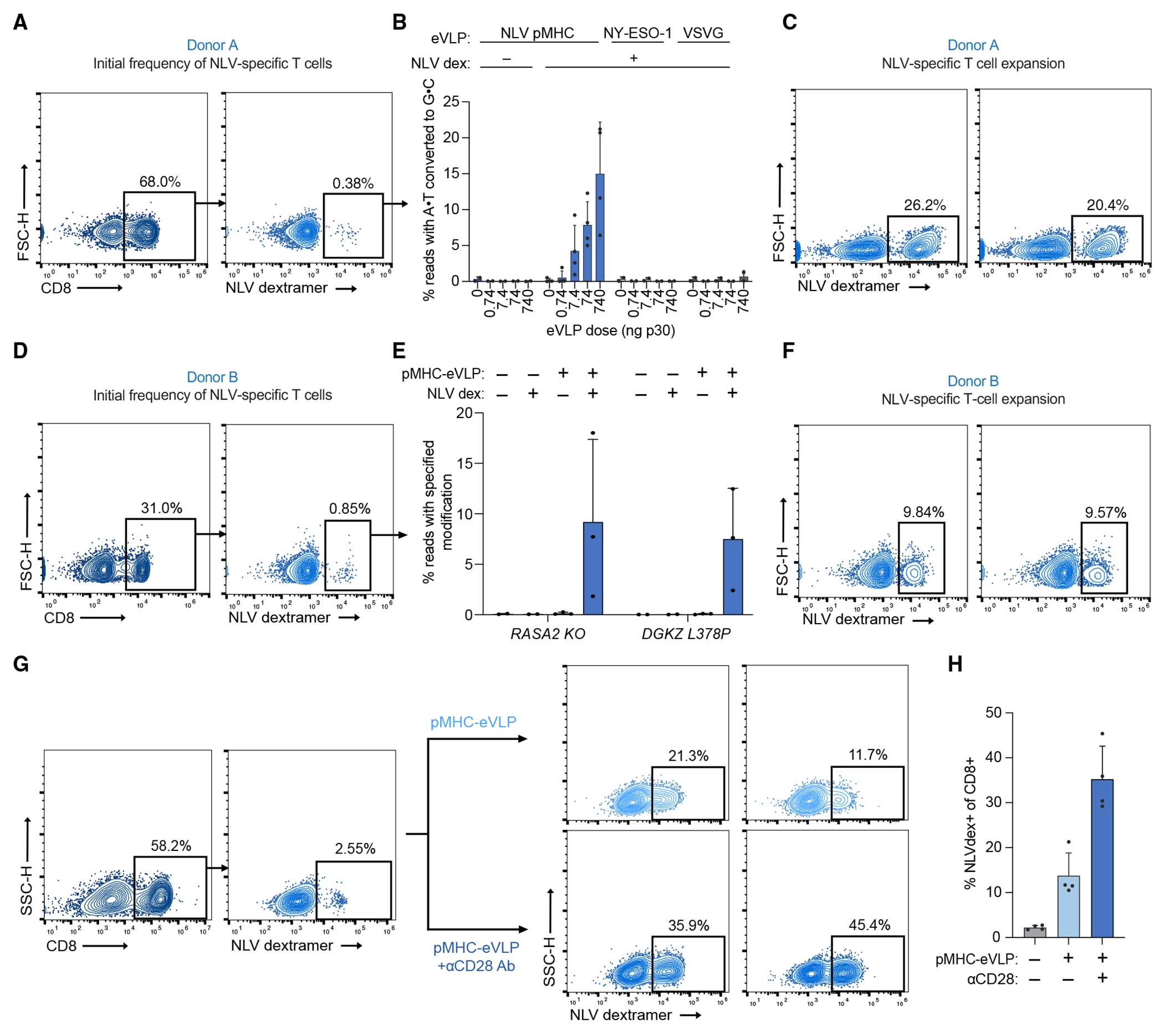
Gene editing and expansion of rare antigen-specific T cells among polyclonal repertoires (A) Representative flow cytometry plots of CD8-positive and NLV dextramer-positive cells in PBMCs from donor A. (B) Base editing efficiencies at *PDCD1* in NLV dextramer-positive and -negative cells from donor A following treatment with the indicated eVLPs. (*n* = 2–4 replicates). (C) Representative flow cytometry plots of NLV dextramer-positive cells among CD8-positive cells from donor A following treatment with NLV pMHC-eVLPs. (D) Representative flow cytometry plots of CD8-positive and NLV dextramer-positive cells in PBMCs from donor B. (E) Gene editing efficiencies at *RASA2* and *DGKZ* in NLV dextramer-positive and -negative cells from donor B following treatment with the indicated eVLPs. (*n* = 2–3 replicates). (F) Representative flow cytometry plots of NLV dextramer-positive cells among CD8-positive cells from donor B following treatment with NLV pMHC-eVLPs. (G) Representative flow cytometry plots of CD8-positive and NLV dextramer-positive cells before and after treatment with NLV pMHC-eVLPs alone or in combination with soluble anti-CD28 antibody. (H) Summary data of NLV-specific CD8^+^ T cell expansion experiment in (G). (*n* = 4 replicates).

We further challenged v.2 pMHC-eVLPs to selectively target rare antigen-specific CD8^+^ T cells within their endogenous primary repertoires. We isolated bulk peripheral blood mononuclear cells (PBMCs) from an HLA-A*02:01-positive human donor and confirmed the presence of rare NLV antigen-specific CD8^+^ T cells using NLV pMHC dextramer staining (Figure 5A). These bulk PBMCs were treated with eVLPs packaging *PDCD1*-ablative ABE8e RNPs pseudotyped with either NLV pMHC, an antigen off-target NY-ESO-1 pMHC, or VSVG. Ten days following eVLP treatment, we used fluorescence-activated cell sorting (FACS) to isolate NLV dextramer-positive and dextramer-negative cells from the bulk PBMC culture for analysis by HTS. Strikingly, we observed efficient *PDCD1* base editing exclusively in NLV-specific CD8^+^ T cells in cultures treated with NLV pMHC-eVLPs but not NY-ESO-1 pMHC-eVLPs or VSVGwt eVLPs, with no detected editing above background in dextramer-negative cells (Figure 5B). This highly selective delivery was consistent across blood donors and different eVLP-encoded gene edits, further confirming the translatability and programmability of the pMHC-eVLP system (Figures 5D and 5E).

Strikingly, we also observed robust expansion of the NLV-specific T cell compartment exclusively in PBMC cultures treated with NLV pMHC-eVLPs across donors, demonstrating that pMHC-eVLPs were capable of selectively activating and expanding antigen-specific T cells in addition to mediating intracellular protein delivery (Figures 5C and 5F). This expansion was not associated with detected increases in the exhaustion profile of the NLV-specific compartment (Figure S5A). Interestingly, we found that this antigen-specific T cell expansion induced by cognate pMHC-eVLP treatment could be significantly augmented by supplemental co-stimulatory signaling, as we observed markedly increased NLV-specific T cell expansion when PBMCs were treated with both NLV pMHC-eVLPs and soluble anti-CD28 antibody compared to pMHC-eVLPs alone (Figures 5G and 5H), indicating that pMHC-eVLPs can be integrated with additional co-stimulatory cues to further enhance antigen-specific T cell priming.

Collectively, these findings establish that the improved v.2 pMHC-eVLP particles can drive highly selective and coordinated priming, expansion, and gene editing of primary antigen-specific CD8^+^ T cells among their endogenous polyclonal repertoires; that pMHC-eVLPs can synergize with co-stimulatory adjuvants to drive antigen-specific T cell expansion from polyclonal repertoires; and that these particles integrate antigen-specific TCR engagement and signaling to mediate cellular entry with precise selectivity.

### pMHC-eVLPs enhance tumor control by polyclonal lymphocytes

Low frequencies and suboptimal phenotypes of tumor-specific T cells often limit the ability of therapeutic TIL products to exert potent and durable anti-tumor responses, necessitating strategies that can selectively expand and enhance the effector phenotypes of minority tumor-specific T cell compartments present in polyclonal TIL expansions. To this end, we hypothesized that pMHC-eVLPs presenting tumor-relevant antigens and encoding immunostimulatory gene edits could be used to enhance the antigen-specific anti-tumor response from an expanded polyclonal lymphocyte culture mimicking a TIL expansion.

**Figure 6.**
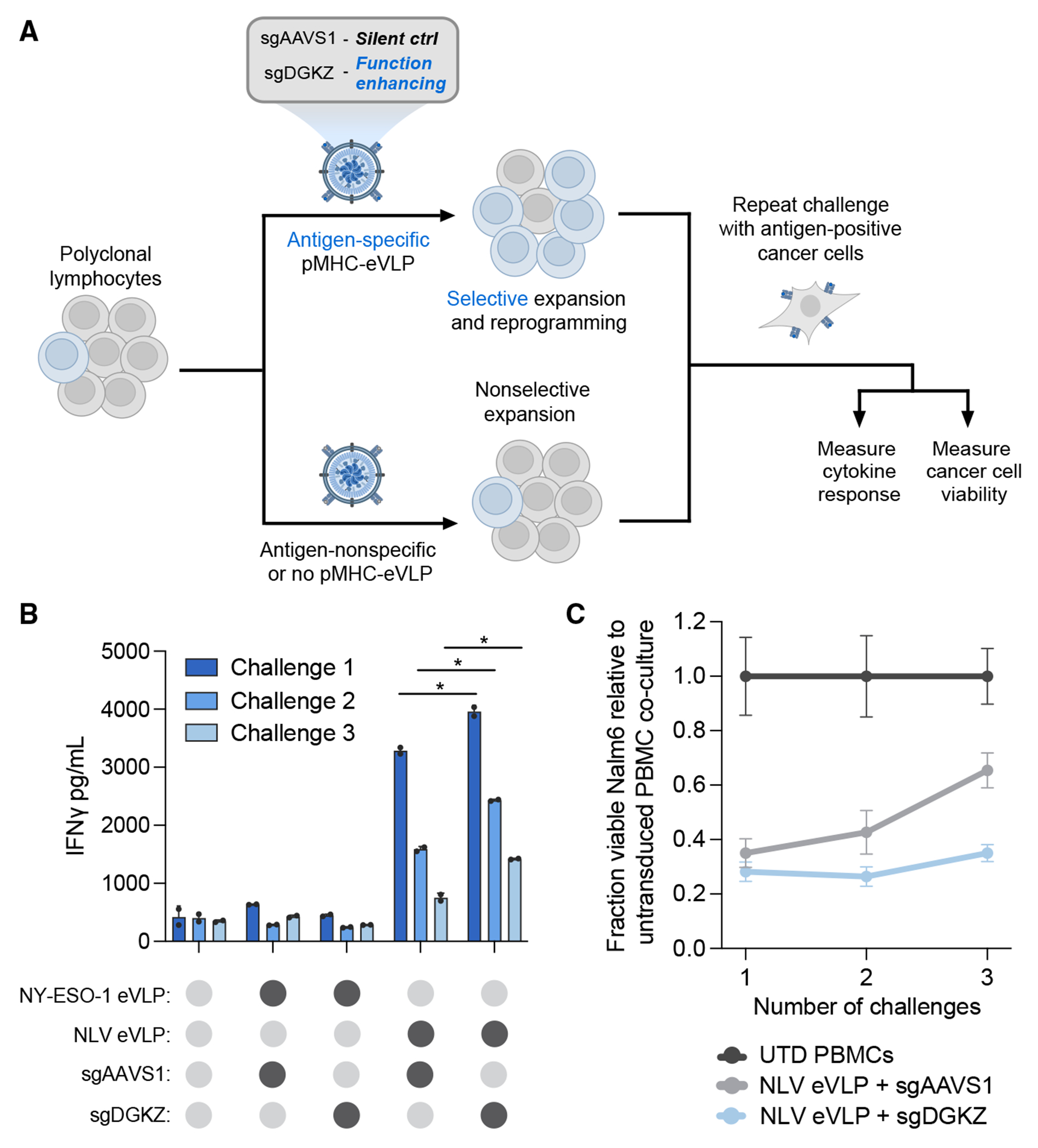
pMHC-eVLPs enhance antigen-specific tumor control by polyclonal lymphocytes (A) Schematic of repeat cancer cell co-culture challenge experiment. (B) Interferon gamma response to Nalm6-NLV cancer cells by PBMCs treated with pMHC-eVLPs measured by ELISA. *p* values calculated using Welch’s t test.*p < 0.05. (*n* = 2 replicates). (C) Viability of Nalm6-NLV cancer cells after co-culture with untransduced expanded PBMCs or PBMCs expanded with treatment of NLV pMHC-eVLPs encoding the indicated edits. PBMCs were challenged repeatedly by serial addition of cancer cells every 48 h. Nalm6 viabilities at each co-culture challenge are normalized to the respective untransduced PBMC co-culture condition. (*n* = 3 replicates).

We first tested this hypothesis by assessing the antigen-specific effector cytokine response of a polyclonal lymphocyte culture following acute challenge with antigen-presenting cells. We treated PBMCs from an HLA-A*02:01 and CMV positive donor with pMHC-eVLPs encoding either a phenotypically silent control base edit (*AAVS1*) or an immunostimulatory base edit (*DGKZ* L378P) presenting either NLV or NY-ESO-1 antigen and repeatedly challenged these PBMCs with Nalm6 cancer cells engineered to express the NLV antigen, quantifying supernatant IFNγ after each co-culture (Figure 6A). We observed significantly higher IFNγ levels produced by PBMCs that had been reprogrammed by NLV pMHC-eVLPs compared to antigen-nonspecific control NY-ESO-1 pMHC-eVLPs and an untreated and unstimulated control (Figure 6B). Consistent with our prior immunostimulatory base editing results and previous reports, PBMCs treated with *DGKZ*-targeted NLV pMHC-eVLPs exhibited additionally increased cytokine response compared to PBMCs treated with *AAVS1*-targeted NLV pMHC-eVLPs (Figure 6B). Together, these data confirmed that pre-treatment of polyclonal lymphocyte repertoires with immunostimulatory pMHC-eVLP particles presenting antigens matched to subsequent antigenic challenge can enhance the antigen-specific effector response.

We next assessed the ability of antigen-specific pMHC-eVLP treatment to enhance the ability of a polyclonal lymphocyte culture to maintain cytotoxic control of antigen-positive cancer cells. We expanded PBMCs from an HLA-A*02:01 and CMV positive donor in the presence of PBS or NLV pMHC-eVLPs encoding either silent control (*AAVS1*) or immunostimulatory (*RASA2* or *DGKZ*) edits. Consistent with previous experiments, treatment with NLV pMHC-eVLPs induced robust expansion of the NLV-specific T cell compartment (Figure S5B). We subsequently challenged these PBMCs with Nalm6 cancer cells engineered to express the NLV antigen, and found that all PBMCs pre-treated with NLV pMHC-eVLPs exhibited strikingly increased cancer killing capacity compared to PBMCs expanded in the absence of pMHC-eVLP simulating a traditional non-selective TIL expansion (Figure 6A).

While all PBMCs treated with NLV pMHC-eVLPs exhibited uniformly enhanced cancer killing activity over non-selectively expanded PBMCs upon first challenge, a cancer killing advantage in *DGKZ* and *RASA2* edited conditions became evident after multiple Nalm6 cancer challenges, consistent with reported effects of these genotypes on T cell persistence and durability.^12,14,16^ In contrast to PBMCs pre-treated with NLV pMHC-eVLPs encoding a phenotypically silent edit at *AAVS*, which exhibited a gradual decline in ability to control cancer cell growth with repeated challenge, PBMCs treated with pMHC-eVLPs encoding *RASA2* ablation or *DGKZ* L378P maintained robust killing capacity after multiple stimulations (Figures 6C and S5C). Similarly, we observed that pMHC-eVLP reprogramming of polyclonal lymphocytes also enhanced cancer cell killing in a solid tumor challenge experiment performed by repeated co-culture with NLV-expressing A375 melanoma cells (Figure S5D).

Collectively, these results demonstrate that antigen-specific pMHC-eVLPs can be used to enhance the anti-tumor potency and durability of polyclonal lymphocyte repertoires by expanding and genetically engineering the tumor-specific T cell compartment.

## DISCUSSION

The clinical successes of existing TIL therapies demonstrate the promise of harnessing anti-tumor T cells present in patients’ endogenous repertoires. To address the therapeutic efficacy limitations imposed by low frequencies and suboptimal phenotypes of tumor-specific T cells often found in TIL products, we developed and optimized pMHC-eVLPs as a novel delivery system that enables highly selective and coordinated priming, expansion, and editing of user-defined antigen-specific CD8^+^ T cell clonotypes among their endogenous polyclonal repertoires while sparing the bystander T cell repertoire. We demonstrate that pMHC-eVLP specificity can be easily programmed with desired pMHC antigens and HLA allotypes to direct specific activity toward antigen-specific T cells via their natural TCRs, and that pMHC-eVLP cargo can be programmed to carry desired CRISPR nuclease, base editor, or prime editor RNPs for efficient editing of endogenous genes. Engineering various aspects of the pMHC-eVLP particle enabled therapeutically relevant *ex vivo* delivery efficiencies in rare antigen-specific T cells among endogenous polyclonal repertoires, and through antigen response and cancer cell killing experiments, we show that optimized pMHC- eVLPs can be used to therapeutically reprogram polyclonal lymphocytes to potentiate robust and durable anti-tumor cytotoxicity.

The single-effector, programmable pMHC-eVLP platform unifies two major therapeutic strategies in cancer immunotherapy: selective expansion and genetic engineering of anti-tumor T cells. Overall, our findings establish pMHC-eVLPs as a novel agent for cancer immunotherapy that enables the use of immunostimulatory genome edits for enhancing TIL therapy without the associated risks of bystander T cell engineering or persistent gene editor expression, which are inherent to existing approaches. To our knowledge, pMHC-eVLPs represent the first use of virus-like particles for antigen-specific human T cell engineering, and provide a complementary strategy to retrovirus-based systems for modulation of antigen-specific T cells and enabling novel T cell engineering applications in which transient protein delivery is necessary for safety over permanent viral DNA integration and constitutive transgene expression.^19,20,30,31^

The pMHC-eVLP platform could be readily applied to existing *ex vivo* TIL manufacturing workflows to enhance the composition and phenotype of *ex vivo* TIL products. Tools to rapidly determine pMHCs relevant in individual patient tumors in real-time already exist and are used for other therapeutic modalities harnessing antigen-specific T cells; these tools could be leveraged to inform pMHC-eVLP treatment strategies. The modularity and generalizability of pMHC-eVLP specificity offer the potential to therapeutically reprogram multiple user-defined T cell clonotypes simultaneously by leveraging pools of pMHC-eVLPs displaying tumor-relevant antigens as a strategy to enhance multi-antigen tumor targeting by TIL products and reducing the potential for immune escape. Successful therapeutic applications of pMHC-eVLPs in TIL therapy may require multiplexed antigen targeting to potentiate poly-antigenic anti-tumor responses, and utilization of multiple public and private antigenic sources including tumor-associated antigens (TAAs, e.g., NY-ESO-1, MART-1), antigens involved in virally driven cancers (e.g., EBV, HPV), and mutation-derived neoantigens. Future therapeutic applications could also explore the use of pMHC-eVLPs directly *in vivo* to reprogram antigen-specific T cells for fully *in situ* cell therapy manufacturing, a strategy that has been demonstrated in recent work with retroviruses and murine T cell repertoires.^29^

In this study, we focused on a small set of previously described immunostimulatory Cas9 nuclease knockouts and precise base edits. However, pMHC-eVLPs could be produced to deliver virtually any gene editing RNP that is compatible with the eVLP vector as well as other protein or RNA cargoes of interest. Here, we showed that pMHC-eVLPs can synergize with CD28-mediated co-stimulation to further enhance antigen-specific T cell expansion. Future work could further explore pMHC-eVLP synergy with other combination treatments, or the addition of co-pseudotypes in combination with pMHC SCTs on the eVLP surface to further modulate target cell specificity or signaling. Because CD8 can cooperatively stabilize class I TCR-pMHC interactions to amplify downstream signaling, we anticipate that co-receptor dependence may influence the efficiency of pMHC-eVLP entry for some TCRs, particularly near the activation threshold. While the present study focuses on class I-restricted targeting of antigen-specific CD8^+^ T cells, future extensions to class II, CD4^+^ settings represent an important direction for future work. Beyond cancer therapy, pMHC-eVLPs could be leveraged to modulate T cell compartments relevant in other disease contexts such as anti-viral cell therapies in settings of immunodeficiency, or self-antigen cross-reactive T cells in T cell-driven autoimmune diseases.

### Limitations of the study

An important limitation to this study and the potential therapeutic application of pMHC-eVLPs is the transduction efficiency observed in polyclonal primary cell settings. While our functional assays suggest that the delivery efficiencies achieved by current pMHC-eVLP formulations are sufficient to drive enhanced antigen responses and tumor control, we anticipate that further engineering of pMHC-eVLPs could yield enhancements that improve their therapeutic potential as a precision TIL reprogramming agent. Future *in vivo* applications of pMHC-eVLPs could also explore potential therapeutic synergy with other immunotherapy modalities such as checkpoint blockade or cancer vaccines to maximize the potential for potent and durable anti-tumor response.

### RESOURCE AVAILABILITY

#### Lead contact

Further information and requests for resources and reagents should be directed to and will be fulfilled by the lead contact, drliu@fas.harvard.edu .

#### Materials availability

Plasmids are available upon request to the lead contact with a completed materials transfer agreement.

## STAR ★ METHODS

### METHOD DETAILS

#### Plasmid cloning and construction

All primers were ordered from Integrated DNA Technologies (IDT) and gene fragments were synthesized by Twist Biosciences and IDT. All constructs were made with Gibson Assembly. The plasmid pCMV-VSV-G was previously developed and published by the Liu Lab (RRID:Addgene_8454). The plasmid pMD2-VSVGmut was previously developed and published by the Birnbaum Lab (RRID:Addgene_182229). The plasmids for eVLP production were previously developed and published by the Liu Lab. sgRNAs were synthesized as gene fragments and cloned into plasmids under the U6 promoter. All spacer sequences used are listed in Table S1. Plasmids to express peptide-MHC single-chain trimers with defined antigens were created as previously described, and consisted of a B2M signal sequence, antigenic peptide, 3xG4S linker, B2M, 4xG4S, and the HLA heavy chain. Single-chain trimer (SCT) point mutants were created using overlapping primers; the MHC groove-opening SCT mutant consisted of an HLA Y84A mutation, and the disulfide-trapped SCT mutant consisted of a Y84C mutation and a G to C mutation in the second residue position of the first G4S linker. T cell receptors were synthesized as gene fragments in the format beta chain, P2A, alpha chain, using murinized constant chains, and cloned into lentiviral transfer plasmids under an EF1a promoter.

#### Lentivirus production

Lentivirus encoding full-length TCRs was produced using HEK293T cells cultured in T75 flask format. Briefly, 5 million producer cells were seeded per flask and transfected the next day with lentiviral transfer plasmid encoding the TCR (9 μg), psPAX2 (9 μg), and pCMV-VSV-G (6 μg) using Lipofectamine 2000 (Thermo Fisher). 40-48 h following transfection, producer cell supernatant was harvested and centrifuged for 5 min at 500 g to remove cell debris and filtered using a 0.45 μm PVDF filter prior to use.

#### Flow cytometry and cell sorting

Flow cytometry was conducted using either CytoFLEX S or CytoFLEX LX Flow Cytometer instruments (Beckman Coulter) at the Broad Technology Space at the Broad Institute. Fluorescence-assisted cell sorting (FACS) was conducted using a Sony MA900 Cell Sorter (Sony Biotechnology) at the Broad Technology Space at the Broad Institute.

For flow cytometry or FACS of cells with surface marker stains, cells were first washed with PBS and then stained with LIVE/DEAD Fixable Viability dye (Invitrogen) for 15 min on ice. Cells were then washed with FACS buffer (PBS with 2% FBS) and then stained for 20 min on ice with fluorescently labeled antibodies. If pMHC dextramers were used, cells were stained in FACS buffer with dextramer for 10 min on ice prior to addition of FACS buffer containing antibody stains followed by an additional 20 min on ice. Cells were then washed once with FACS buffer prior to resuspension in FACS buffer for acquisition. For intracellular staining, cells were prepared using the Cyto-Fast Fix/Perm Buffer Set (BioLegend) according to manufacturer protocols.

#### eVLP production, purification, and quantification

BE-eVLPs were produced as previously described. Briefly, Gesicle Producer 293T cells were seeded in T75 flasks at a density of 5 million cells per flask. After 20–24 h, cells were transfected using the jetPRIME transfection reagent (Polyplus) according to the manufacturer’s protocols. For producing VSVGwt-pseudotyped eVLPs, a mixture of plasmids expressing VSV-G (400 ng), MMLVgag^Q226^-pro-pol (2800 ng), MMLVgag^C507^-ABE8e (1700 ng), and sgRNA (4500 ng) were co-transfected per T75 flask. For producing optimized pMHC-eVLPs, cells were transfected with a mixture of plasmids expressing pMHC SCT (900 ng), VSVGmut (300 ng), MMLVgag ^Q226P^-pro-pol (675 ng), MMLV_Pr65_gag ^Q226P^-pro-pol (2700 ng), MMLV_gPr80_gag^C507V^-ABE8e (1125 ng), and sgRNA (4500 ng).

40-48 h following transfection, producer cell supernatant was harvested and centrifuged for 5 min at 500 g to remove cell debris and filtered using a 0.45 μm PVDF filter. For all eVLPs used in cell line experiments, unless otherwise stated, the filtered supernatant was concentrated 50-fold using PEG-it Virus Precipitation Solution (System Biosciences) according to the manufacturer’s protocols. For all eVLPs used in primary cell experiments, unless otherwise stated, filtered supernatant was concentrated 1000-fold by ultracentrifugation using a 20% (w/v) sucrose cushion in PBS. Ultracentrifugation was performed at 26,000 rpm at 4°C for 30 min to 2 h using an SW28 rotor in an Optima XPN Ultracentrifuge (Beckman Coulter). Following ultracentrifugation, eVLPs were resuspended in PBS overnight at 4°C and subsequently frozen and stored at −80°C. eVLP titers were quantified for normalizing primary cell transduction experiments via MLV p30 mass per volume using the MuLV Core Antigen ELISA Kit (Cell Biolabs, VPK-156) following the manufacturer protocols.

#### eVLP transduction and genomic DNA collection

For experiments involving pMHC-eVLP transduction of Jurkat cells, target cells were plated at a density of 20,000 cells per well in round-bottom 96-well plates (Corning) in 100 μL media volume. Immediately following plating, pMHC-eVLPs were added to wells at the indicated doses. An equivalent volume of plating media was added 24 h after transduction. Genomic DNA was harvested 72 h after transduction; briefly, cells were pelleted and washed once with 1X PBS using centrifugation steps (5 min, 300 g) before resuspension in 50 μL of lysis buffer (10 mM Tris-HCl pH 8.0, 0.05% SDS, 25 μg mL^−1^ proteinase K). Cells were lysed for 1–2 h at 37°C and then heated to 80°C for 30 min and used as direct input for downstream HTS preparation as described below.

For experiments involving VSVGwt-pseudotyped eVLP transduction of primary T cells, target T cells were thawed and rested overnight in media containing IL-2 as described above at a density of 1e6 cells per mL. 20–24 h after thaw, T cells were activated using ImmunoCult Human CD3/CD28/CD2 T cell Activator (StemCell Technologies) according to manufacturer protocols. 48–72 h after activation, target cells were plated at a density of 20,000 cells per well in round-bottom 96-well plates (Corning) in 100 μL media volume. Immediately following plating, eVLPs were added to wells at the indicated doses. Cells were maintained with half-media exchanges every 48 h. Genomic DNA was harvested 7–8 days after transduction according to the same protocol described above.

For experiments involving pMHC-eVLP transduction of primary TCR-T cells, T cells were first thawed and rested overnight in media containing IL-2 as described above at a density of 1e6 cells per mL. 20–24 h after thaw, T cells were activated using Dynabeads Human T-Activator CD3/CD28 (Thermo Fisher) according to manufacturer protocols. 48 h following activation, T cells were transduced with lentivirus encoding full-length TCRs using spinoculation (centrifugation for 2 h at 1000 g at 37°C). 24 h following lentiviral transduction, activator beads were removed using a magnet and resuspended in fresh media. 72 h after bead removal, T cells expressing exogenous TCRs were isolated using FACS for murine TCR beta chain-positive cells. Isolated TCR-T cells were rested for a minimum of one week following sorting prior to subsequent experimentation. Target TCR-T cells were subsequently plated at a density of 20,000 cells per well in round-bottom 96-well plates (Corning) in 100 μL media volume. Immediately following plating, eVLPs were added to wells at the indicated doses. Cells were maintained with half-media exchanges every 48 h. Genomic DNA was harvested 7–8 days after transduction according to the same protocol described above.

For experiments involving pMHC-eVLP transduction of primary PBMCs, cells were first thawed and rested overnight in media in the absence of IL-2 at a density of 1e6 cells per mL. 20–24 h after thaw, PBMCs were resuspended in fresh media containing IL-2 and plated in flat-bottom 96-well plates (Corning) at a density of 100,000 to 200,000 cells per well in a media volume equating to 1e6 cells per mL. Immediately following plating, pMHC-eVLPs were added to wells at the indicated doses. Cells were maintained with half-media exchanges every 48 h 10–14 days following transduction, cells were prepared for FACS, and dextramer-positive and dextramer-negative CD8^+^ cell populations were isolated. Genomic DNA was purified from sorted cell samples using the Qiagen DNeasy Kit according to manufacturer protocols.

#### Intracellular flow cytometry following eVLP transduction

Primary T cells were treated with eVLPs as described above and cultured for 7–10 days following transduction before subsequent experiments. For intracellular cytokine analysis, cells were stimulated with ImmunoCult as described above with eBioscience 1X Protein Transport Inhibitor Cocktail (Thermo Fisher). 6–9 h after stimulation, cells were prepared for intracellular marker flow cytometry as described above. Intracellular cytokine stains were used at a 1:100 dilution. For intracellular phosphoprotein analysis, cells were stimulated with ImmunoCult for a predetermined time interval. Following stimulation, cells were immediately fixed by adding an equal volume of pre-warmed BD Phosflow Fix Buffer I (BD) for 10 min at 37°C. Cells were then washed once with FACS buffer and then permeabilized by adding BD Phosflow Perm Buffer III and incubated for 30 min at −20°C. Cells were then washed twice and stained at room temperature in the dark followed by two washes prior to acquisition. Intracellular phosphoprotein stains were used at a 1:50 dilution.

#### High-throughput sequencing of genomic DNA

HTS was performed as previously described. Primers used for the amplification of genomic loci and corresponding amplicons are listed in Table S2. Briefly, 1–5 μL of cell lysate or 100–200 ng of purified genomic DNA as described above was used directly for amplification of the target locus using a first round of PCR (PCR1) using Phusion U Green Multiplex PCR Master Mix (Thermo Fisher) under the following conditions: 98°C (2 min); 25 cycles of 98°C (10 s), 63°C (30 s), and 72°C (45 s); and 72°C (5 min). Subsequently, 1–2 μL of PCR1 product was used as a template for a second round of PCR (PCR2) to append amplicons from unique experimental sample conditions with unique Illumina barcodes. PCR2 was conducted using Phusion U Green Multiplex PCR Master Mix under the following conditions: 98°C (2 min); 10 cycles of 98°C (10 s), 63°C (30 s), and 72°C (45 s); and 72°C (5 min). PCR2 products were pooled and purified on a 1.5% agarose gel by gel extraction using QIAquick Gel Extraction Kit (Qiagen). Libraries were quantified by Qubit dsDNA HS Assay (Thermo Fisher) and were sequenced via Illumina MiSeq 300 v2 Kit (Illumina) on Illumina MiSeq instruments.

#### Cytokine ELISA

PBMCs isolated from HLA-A*02:01 positive and CMV positive healthy donors were treated with pMHC-eVLPs as described above. 10 days following transduction, PBMCs were PBS washed and resuspended in cytokine-free media and serially co-cultured with Nalm6-NLV cells at 72 h intervals via PBS wash and full media exchange. Supernatant was harvested 12 h following each co-culture and cytokine levels were quantified by ELISA MAX Deluxe Set Human (BioLegend).

#### *In vitro* cancer killing assay by pMHC-eVLP treated PBMCs

PBMCs isolated from HLA-A*02:01 positive and CMV positive healthy donors were treated with pMHC-eVLPs as described above. For Nalm6 co-culture experiments, 14 days following transduction, PBMCs were PBS washed and resuspended in cytokine-free media and plated into a flat-bottom 96-well plate at a density of 10,000 cells per well in 100 μL of media. Nalm6 or Nalm6-NLV cells were then transferred into the well at the indicated numbers in 100 μL of media. For serial challenges, more cancer cells were added into the co-culture wells at the indicated numbers via half media exchange. BrightGlo Luciferase Assay System (Promega) was used according to manufacturer protocols to quantify cancer cells at the indicated days following cancer challenge. For A375 co-culture experiments, A375-NLV cells were seeded into flat-bottom 96-well plates at a density of 10,000 cells per well in 100 μL media the day before co-culture. The next day, media was aspirated and PBMCs in 100 μL IL-2 free media were transferred into A375-containing wells. For serial challenges, PBMCs were transferred into new wells containing A375 target cells seeded the day before. Incucyte live-cell imaging was used to continuously quantify cancer cell survival and growth following each co-culture challenge.

#### EXPERIMENTAL MODEL AND STUDY PARTICIPANT DETAILS

##### Cell culture of cell lines and primary human lymphocytes

HEK293T cells (ATCC; CRL-3216), Gesicle Producer 293T cells (Takara; 632617), A375 cells (ATCC; CRL-1619), and A375-NLV-mScarlet cells (generated in this study) were maintained in DMEM + GlutaMAX (Life Technologies) supplemented with 10% fetal bovine serum (FBS). Jurkat (ATCC TIB-152) cells, Nalm6 (ATCC; CRL-3273), and Nalm6-NLV-mScarlet (generated in this study) were cultured in RPMI-1640 supplemented with 10% FBS. T2 cells (ATCC; CRL-1992) were maintained in IMDM (Life Technologies) supplemented with 20% FBS. Primary human T cells and peripheral blood mononuclear cells were commercially sourced de-identified donor cells, confirmed to be HLA-A*02:01 positive and CMV positive by the vendor as needed for experiments, purchased from StemCell Technologies and maintained in RPMI-1640 + GlutaMAX (Life Technologies), 1% penicillin and streptomycin (Thermo Fisher), 1X Nonessential Amino Acids (Thermo Fisher), and 100 IU recombinant human IL-2 (PeproTech). All cells were cultured at 37°C with 5% carbon dioxide and were confirmed to be negative for mycoplasma by testing with MycoAlert (Lonza Biologics). Cell cryopreservation was performed using Bambanker (Bulldog Bio) serum-free cell freezing medium or FBS with 10% DMSO.

Briefly, Jurkat cell lines stably expressing exogenous TCRs were generated using the following method. A TCR-knockout Jurkat line was first created by electroporating wild-type Jurkat cells with plasmids encoding CRISPR Cas9 nuclease and an sgRNA targeting *TRAC* via the Neon electroporation system (ThermoFisher), followed by FACS for TCR-negative cells. Next, TCR-knockout Jurkat cells were transduced with fresh lentivirus encoding full-length TCRs bearing murinized constant beta and alpha chains, followed by FACS to isolate murine TCR beta chain-positive cells.

#### QUANTIFICATION AND STATISTICAL ANALYSIS

Data are presented as mean and standard error of the mean (SEM). No statistical methods were used to predetermine sample size. Quantification and statistical analysis was performed using GraphPad Prism software. Statistical tests used are described in the figure legends. Experiments were performed with three replicates unless otherwise indicated by figure legends or by individually plotted data points.

## Supplementary Material

1

Supporting material can be found online at https://doi.org/10.1016/j.celrep.2026.117510 .

## Figures and Tables

**Table:** KEY RESOURCES TABLE

REAGENT or RESOURCE	SOURCE	IDENTIFIER
Antibodies		
anti-mouse TCR B	Biolegend	Cat#109207, RRID: AB_313430
anti-human B2M	Biolegend	Cat# 395711, RRID: AB_2801060
anti-human CD8	Biolegend	Cat#344717, RRID: AB_10551616
anti-human CD3	Biolegend	Cat#317305, RRID: AB_571906
anti-human TCR A/B	Biolegend	Cat#306715, RRID: AB_1953256
anti-human CD69	Biolegend	Cat#388603, RRID: AB_3083391
anti-human TNFa	Biolegend	Cat#502908, RRID: AB_315260
anti-human IFNγ	Biolegend	Cat#502511, RRID: AB_315236
anti-human IL-2	Biolegend	Cat#310605, RRID: AB_3083299
anti-human ERK1/2 Phospho (Thr202/Tyr204)	Biolegend	Cat#369505, RRID: AB_2629704
anti-human PD1	Biolegend	Cat#379207, RRID: AB_2922606
HLA-A*0201 (NLVPMVATV) MHC Dextramer	Immudex	Cat#WB02132
Bacterial and virus strains		
One Shot Mach1 T1 Phage-Resistant Chemically Competent E.coli	Thermo Fisher Scientific	Cat#C862003
Chemicals, peptides, and recombinant proteins		
DpnI	New England BioLabs	Cat#R0176S
Lipofectamine 2000	Thermo Fisher Scientific	Cat#11668019
jetPRIME Transfection Reagent	Polyplus	Cat#114-75
FuGENE HD Transfection Reagent	Promega	Cat#E2312
PEG-it Virus Precipitation Solution	System Biosciences	Cat#LV825A-1
Proteinase K	Thermo Fisher Scientific	Cat#EO0492
Recombinant Human IL-2	Peprotech	Cat#200-02
Dynabeads Human T-Expander CD3/CD28 beads	Thermo Fisher Scientific	Cat#1161D
ImmunoCult Human CD3/CD28 T cell Activator	STEMCELL	Cat#10971
CellTrace Violet dye	Thermo Fisher Scientific	Cat#C34571
LIVE/DEAD Fixable Dead Cell Stain	Invitrogen	Cat#L34975
Critical commercial assays		
Phusion U Multiplex PCR Master Mix	Thermo Fisher Scientific	Cat#F562L
QIAquick PCR Purification Kit	QIAGEN	Cat#28104
QIAquick Gel Extraction Kit	QIAGEN	Cat#28704
QIAGEN Plasmid Plus Midi Kit	QIAGEN	Cat#12943
QIAGEN Plasmid Plus Maxi Kit	QIAGEN	Cat#12963
MuLV Core Antigen ELISA Kit	Cell Biolabs	Cat#VPK-156
ONE-Glo Luciferase Assay System	Promega	Cat#E6110
MiSeq Reagent Kit v2 (300-cycles)	Illumina	Cat#MS-102-2002
MiSeq Reagent Micro Kit v2 (300-cycles)	Illumina	Cat#MS-103-1002
NEBuilder HiFi DNA Assembly Master Mix	NEB	Cat# E2621L
ELISA MAX Deluxe Set Human IFN-gamma	Biolegend	Cat#430104
Nunc MaxiSorp ELISA Plates, Uncoated	Biolegend	Cat#423501
Stop Solution for TMB Substrate	Biolegend	Cat#423001
Experimental models: Cell lines		
HEK293T	ATCC	Cat#CRL-3216
Gesicle Producer 293T	Takara	Cat#632617
Jurkat	ATCC	Cat#TIB-152
TCR-expressing Jurkats	Generated in this study.	N/A
Antigen and fLux-mScarlet-expressing Nalm6	Generated in this study.	N/A
Antigen and fLux-mScarlet-expressing A375	Generated in this study.	N/A
Biological samples		
Human Peripheral Blood Pan-T Cells	STEMCELL	Cat#70024
Human Peripheral Blood Mononuclear Cells	STEMCELL	Cat#70025
Oligonucleotides		
Cas9 spacer sequences	Generated in this study.	Table S1.
Primers for HTS preparation.	Generated in this study.	Table S1.
Recombinant DNA		
pCMV-VSVG	Addgene	8454
pMD2.G-VSVG-mut	Addgene	182229
psPAX2	Addgene	12260
pMHC-eVLPs	Generated in this study.	N/A
TCR transfer vectors	Generated in this study.	N/A
Software and algorithms		
CRISPResso2	(Clement et al., 2019)	https://github.com/pinellolab/CRISPResso2
Prism	GraphPad	https://www.graphpad.com
Deposited Data		
Targeted amplicon sequencing data	This study	PRJNA1460378

## Data Availability

The sequencing data generated during this study are available at the NCBI Sequencing Read Archive database under PRJNA1460378. The code used for data processing and analysis are available at https://github.com/pinellolab/CRISPResso2.

## Highlights

- eVLPs enhance T cell function via immunostimulatory genome editing
- Class I pMHC pseudotyping enables selective targeting of antigen-specific CD8^+^ T cells
- pMHC-eVLPs can simultaneously prime, expand, and gene edit antigen-specific CD8^+^ T cells
- pMHC-eVLP T cell reprogramming enhances potency and durability of anti-tumor T cells
