# Supplementary material for: Peptide-MHC-targeted engineered virus-like particles enable selective priming and gene editing of tumor-specific T cells
Source: Cell Rep. Author manuscript; Available in PMC 2026 Jul 31. (PMC13426065; doi:10.1016/j.celrep.2026.117510)
Supplement: 1 [file NIHMS2190750-supplement-1.pdf]

Cell Reports, Volume *45*

Supplemental information

Peptide-MHC-targeted engineered virus-like particles enable selective priming and gene editing of tumor-specific T cells

Brian H. Shim, Q. Henry Zhao, Jack A. Queenan, Blake E. Smith, Michael E. Birnbaum, and David R. Liu

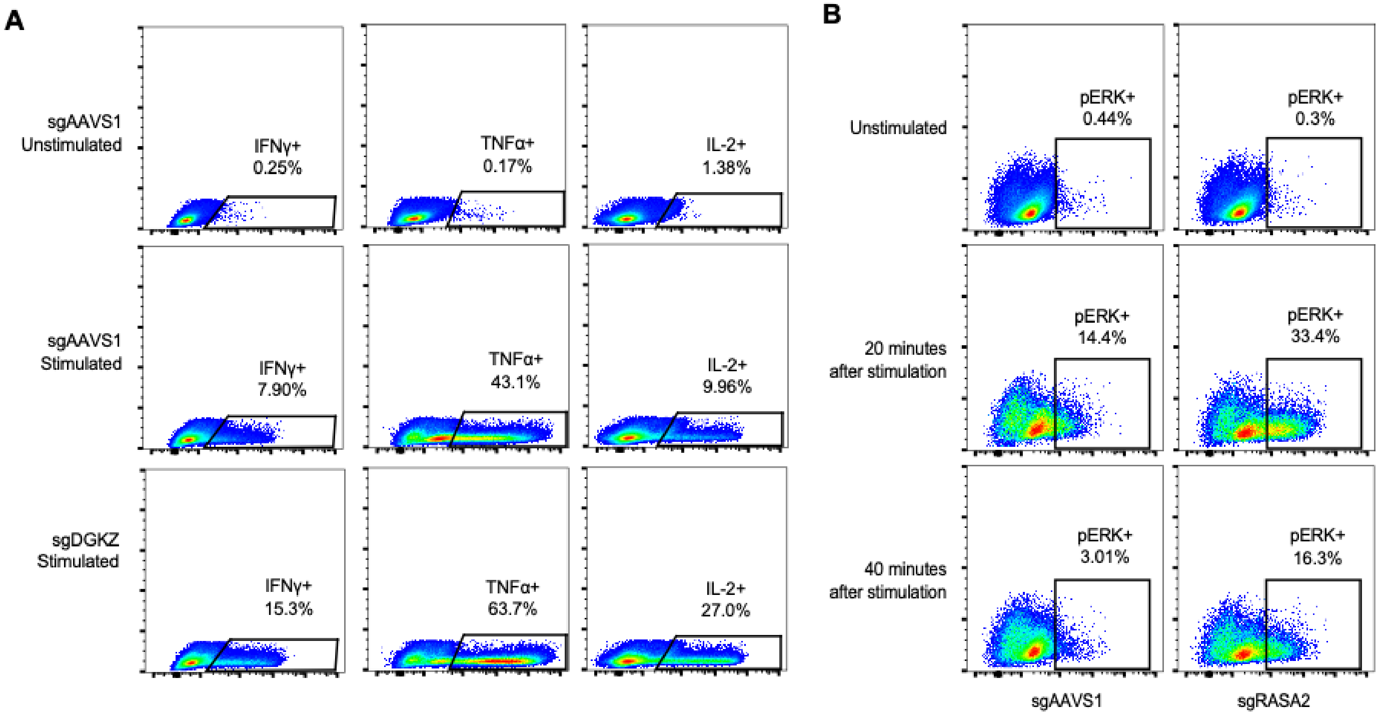

Supplementary Figure 1: Primary human T cells edited with eVLPs display enhanced antigen response.
(A) Representative flow cytometry plots of primary T cells edited with the indicated sgRNAs stained with the indicated intracellular stains following stimulation.
(B) Representative flow cytometry plots of primary T cells edited with the indicated sgRNAs stained with the indicated intracellular stains at the indicated time intervals following stimulation.

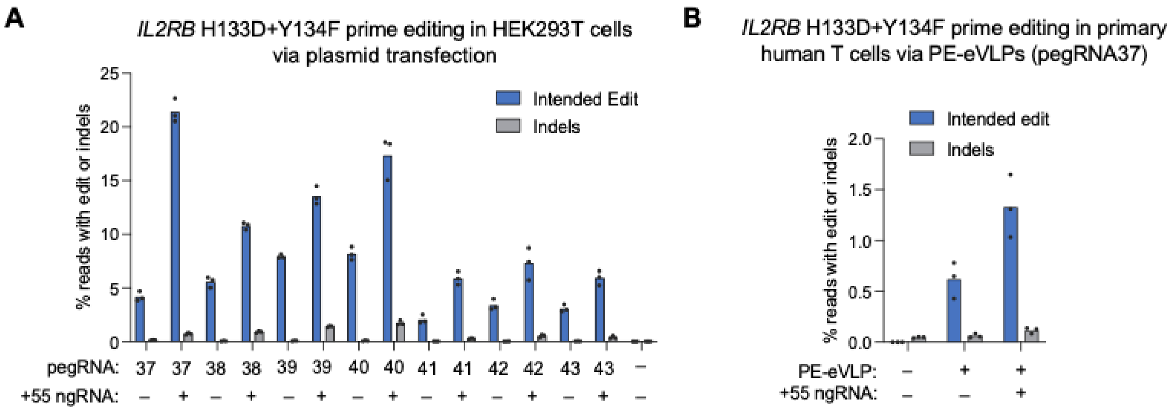

Supplementary Figure 2: Prime editing for *ortho*IL-2 receptor conversion.
(A) Prime editing and indel frequencies in HEK293T cells transfected with plasmids encoding PEmax+SSB and pegRNA and nicking guide RNA (ngRNA) combinations. (*n*=3 replicates).
(B) Prime editing and indel frequencies in primary human T cells transduced with PE-eVLPs packaging PEmax+SSB and pegBHS37 with or without an ngRNA. (*n*=3 replicates).

A

TCR	Ag origin	Epitope	HLA allele	CDR3 beta/alpha
C7	CMV	NLVPMVATV	A*02:01	CASSQTQLWETQYF / CAFITGNQFYF
D1.6	Influenza	GILGFVFTL	A*02:01	CASSIFGGKEQF / CALSEAGTGGSYIPTF
D2.4	EBV	GLCTLVAML	A*02:01	CASSSGTSGYYNEQF/ CVVNKPNDYKLSF

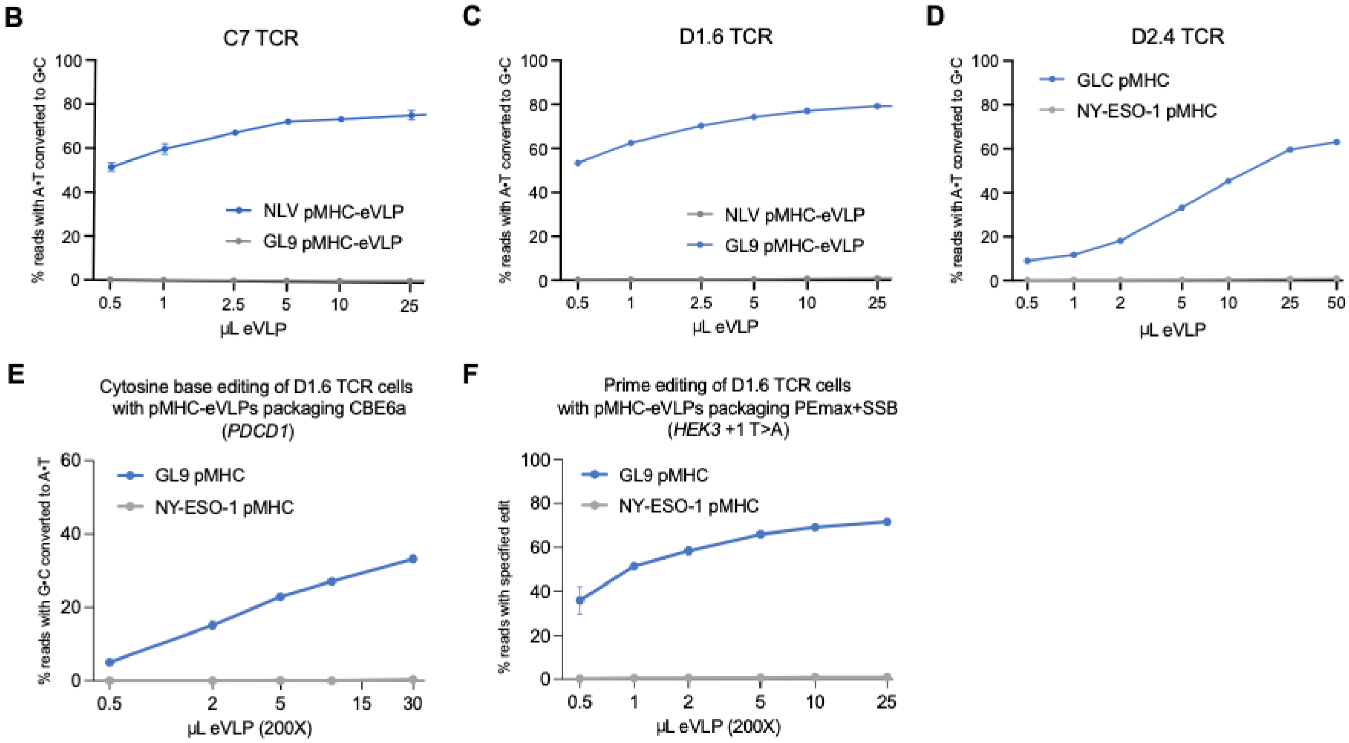

Supplementary Figure 3: Targeted gene editing of antigen-specific T cells with pMHC-eVLPs.
(A) Table of viral antigens, HLA alleles, and cognate TCRs used.
(B-D) Base editing efficiencies in anti-viral TCR cells by pMHC-eVLPs displaying cognate viral or off-target control antigens. (*n*=3 replicates).
(E) Cytosine base editing efficiencies in D1.6 TCR cells by pMHC-eVLPs packaging CBE6a. (*n*=3 replicates).
(F) Prime editing efficiencies in D1.6 TCR cells by pMHC-eVLPs packaging PEmax+SSB. (*n*=3 replicates).

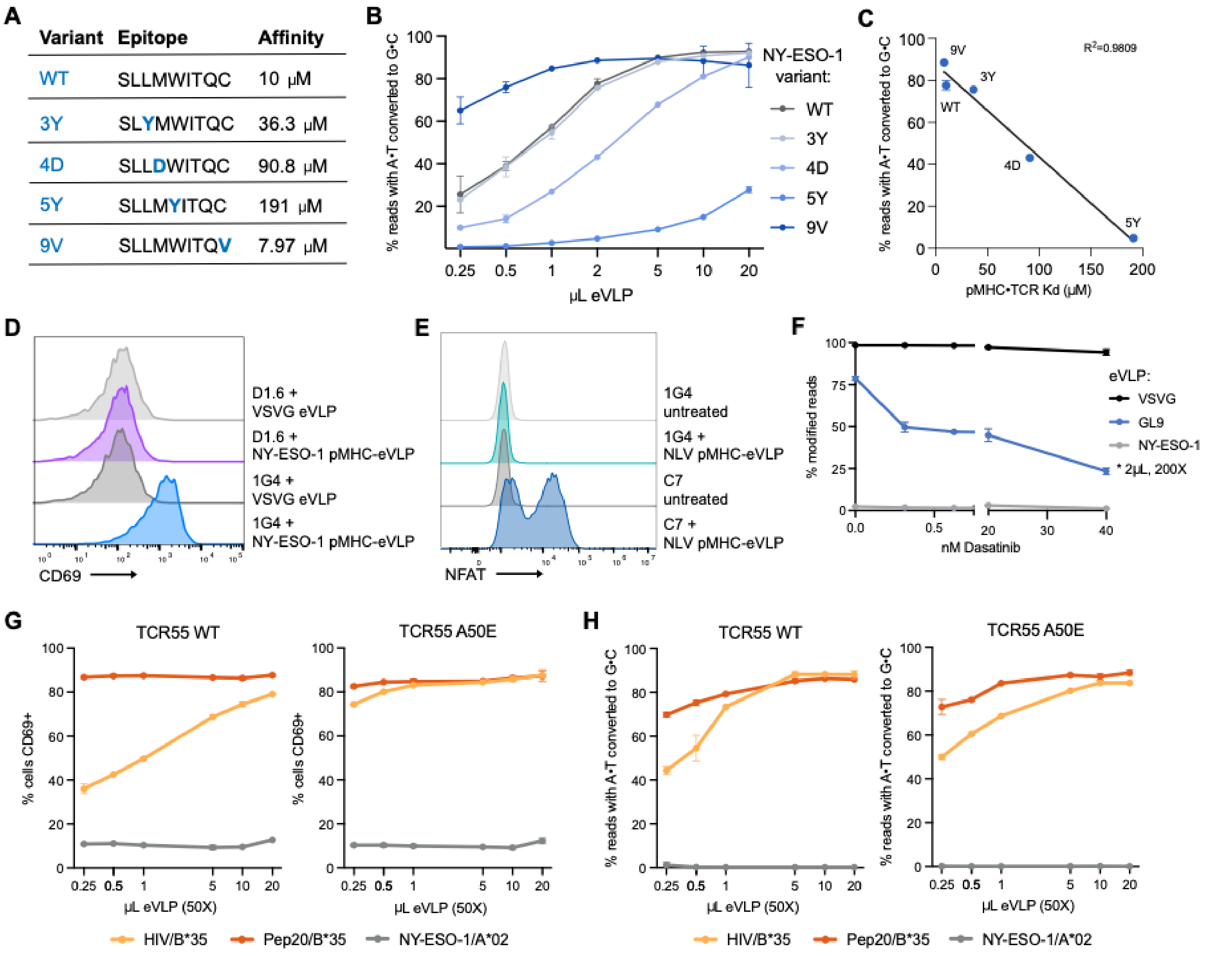

Supplementary Figure 4: pMHC-TCR affinity and TCR activation influence pMHC-eVLP transduction.
(A) Table of NY-ESO-1 variant epitopes and their affinities for the 1G4 TCR.
(B) Base editing efficiencies in 1G4 TCR-expressing cells by pMHC-eVLPs displaying NY-ESO-1 variant epitopes. (*n*=3 replicates).
(C) Correlation of base editing efficiency and pMHC-TCR affinity in 1G4 TCR-expressing cells by pMHC-eVLPs displaying NY-ESO-1 variant epitopes.
(D) Representative flow cytometry histograms of CD69 expression on TCR-expressing Jurkat cells twenty-four hours following treatment by 1 μL of 50X concentrated eVLPs.
(E) Representative flow cytometry histograms of NFAT expression in TCR-expressing Jurkat cells twenty-four hours following treatment by 1 μL of 50X concentrated eVLPs.
(F) Cas9 nuclease editing efficiency in 1G4 TCR cells following treatment by the indicated eVLPs in the presence of varying concentrations of dasatinib. (*n*=3 replicates).
(G) CD69 upregulation in TCR55 or TCR55-A50E-expressing Jurkat cells twenty-four hours following treatment by pMHC-eVLPs. (*n*=3 replicates).
(H) Base editing efficiencies in TCR55 or TCR55-A50E-expressing Jurkat cells by pMHC-eVLPs. (*n*=3 replicates).

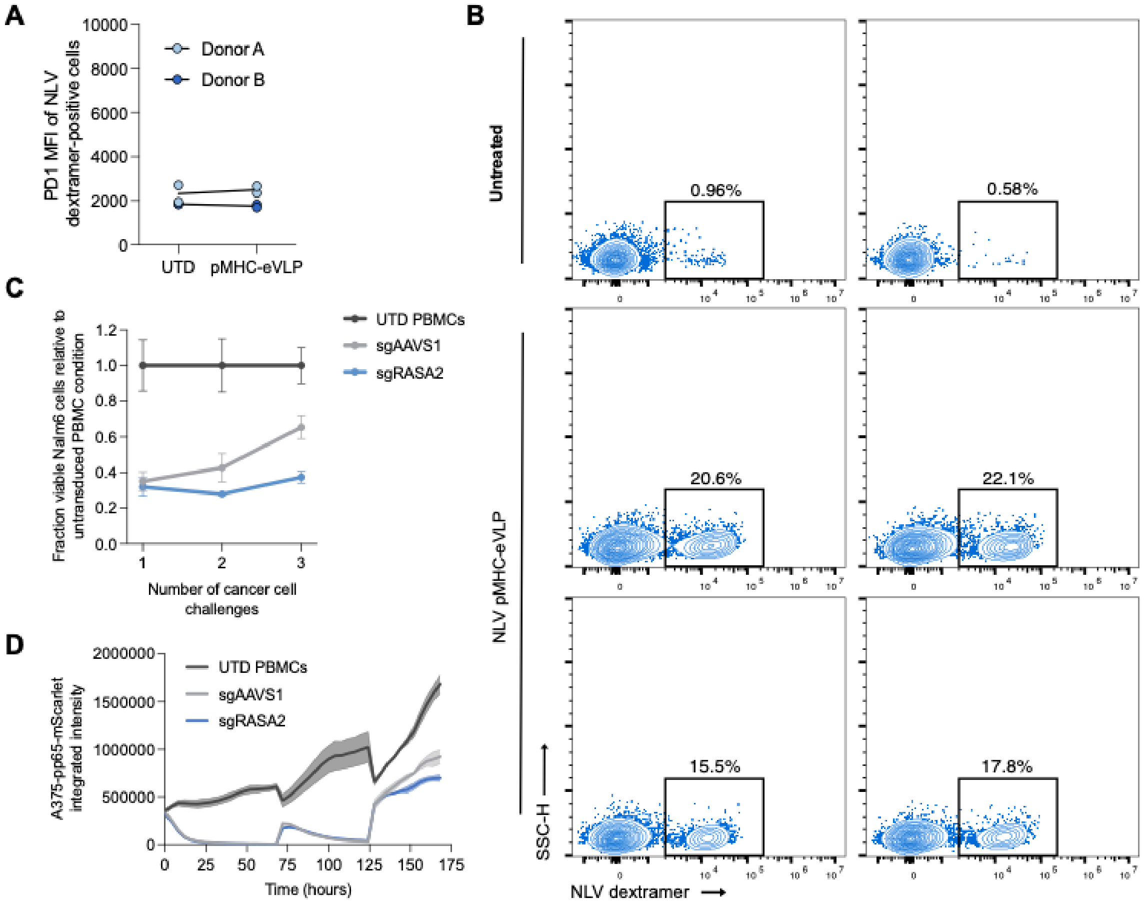

Supplementary Figure 5: pMHC-eVLPs enhance the antigen-specific composition and anti-tumor response of polyclonal lymphocytes.

(A) Mean fluorescence intensity of PD1 measured by flow cytometry in NLV dextramer-positive CD8 T cells from donors A and B 14 days following treatment by PBS or NLV pMHC-eVLPs. (*n*=2 replicates).

(B) Representative flow cytometry plots of NLV dextramer-positive CD3-positive cells 13 days following treatment with PBS or NLV pMHC-eVLPs before use in cancer cell co-culture experiments (Fig. 6C-D, Fig. S6B).

(C) Viability of Nalm6-NLV cancer cells after co-culture with untransduced expanded PBMCs or PBMCs expanded in the presence of NLV pMHC-eVLPs encoding the indicated edits. PBMCs were challenged repeatedly by serial addition of cancer cells every 48 hours. Nalm6 viabilities at each co-culture challenge are normalized to the respective untransduced PBMC co-culture condition. (*n*=3 replicates).

(D) Incucyte tracking of mScarlet integrated fluorescence intensity in wells co-culturing A375-NLV-mScarlet with PBMCs pre-treated with PBS, sgAAVS1 NLV pMHC-eVLPs, or sgRASA2 NLV pMHC-eVLPs. PBMCs were re-challenged at hours 75 and 125 for a total of three serial A375-NLV-mScarlet co-cultures. (*n*=2 replicates).

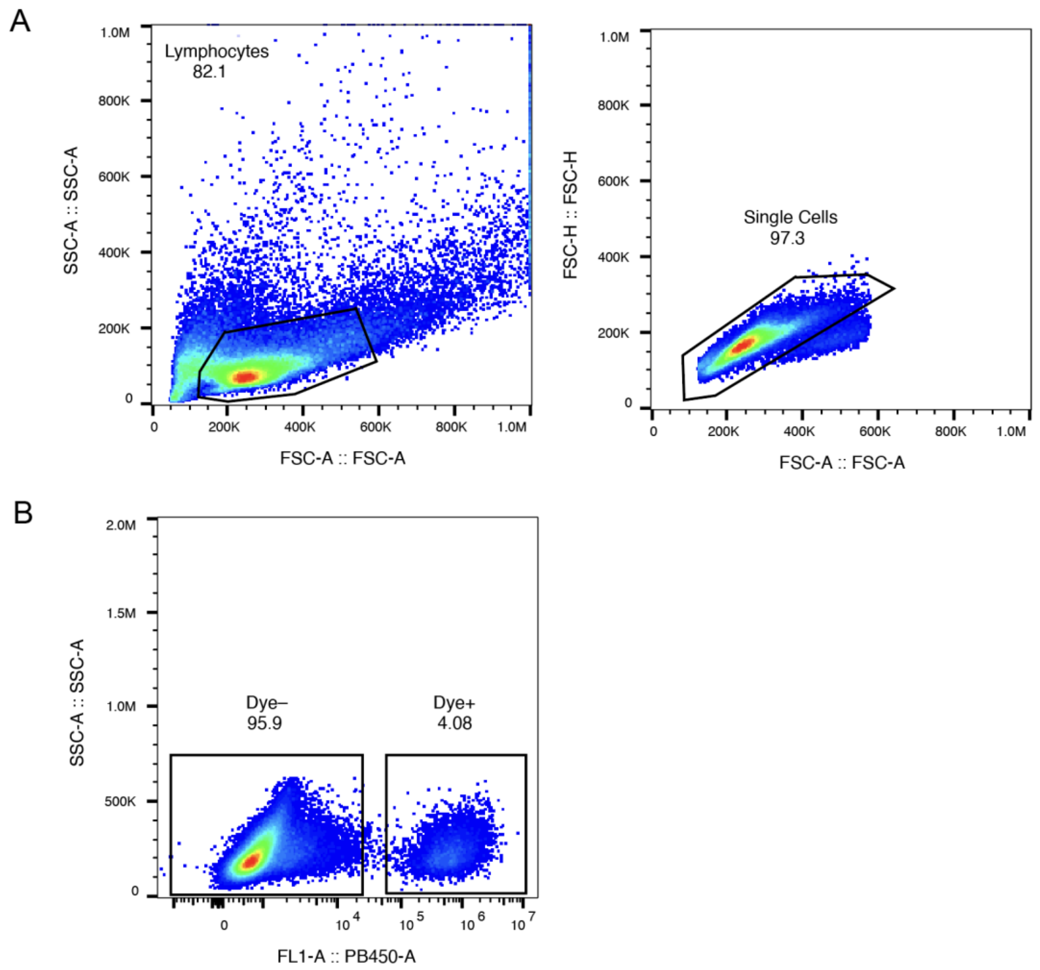

Supplementary Note 1: Example gating strategy for flow cytometry and fluorescence-assisted cell sorting. For experiments involving intracellular cytokine and phosphoprotein stains, cells were pre-gated by size (FSC-A:SSC-A) and singlets (FSC-A:FSC-H or SSC-A:SSC-H) prior to analysis of intracellular staining (see Figure 1 and Extended Data Figure 1). For experiments analyzing mixed TCR-expressing Jurkat cells for B2M expression as a measure of eVLP transduction, cells were pre-gated by size (FSC-A:SSC-A) and singlets (FSC-A:FSC-H or SSC-A:SSC-H) prior to analysis of cell-tracking dye and B2M staining (see Figure 2). For analysis or sorting of antigen-specific CD8+ T cells, cells were pre-gated by size (FSC-A:SSC-A) and singlets (FSC-A:FSC-H or SSC-A:SSC-H), followed by analysis of CD8 and pMHC dextramer staining (see Figure 5 and Extended Data Figure 6). **(A)** Example pre-gating strategy (size and singlets) for all flow cytometry and FACS experiments. **(B)** Example of gating strategy for cell-tracking dye.

Supplementary Table 1: SpCas9 protospacer sequences.

Target locus	Protospacer Sequence
PDCD1	CACCTACCTAAGAACCATCC
B2M	ACTCACGCTGGATAGCCTCC
DGKZ	AGGGGCAGGATGGCAACAGG
PIK3CD	GGGCAGTCCTGCAGAAGGAC
RASA2	AGATATCACACATTACAGTG
TRAC	GGATTTAGAGTCTCTCAGC

Supplementary Table 2: Primer sequences used for high-throughput sequencing.

Target	Forward Primer	Reverse Primer
PDCD1	GACCTGCCAGGGACTGAGGG	GAGAAGGCGGCACTCTGGTG
B2M	GGCTGGGCACGCGTTTAAT	TTGGAGAAGGGAAGTCACGGA
DGKZ	CTGATTTGCCTCTGTTCTTCCTC	CTCTGTGGCTGGAGAGGC
PIK3CD	AGGGCAGGGAAGCTGGGTCT	TGGGACGGAGGGCCACATG
RASA2	AATCCTGCAGGAGAATCTGCG	ACTTACTAGGAAATCTCTGAGTAGCC
